# Genetic and physiological traits for internal phosphorus utilization efficiency in rice

**DOI:** 10.1371/journal.pone.0241842

**Published:** 2020-11-05

**Authors:** Getnet Dino Adem, Yoshiaki Ueda, Patrick Enrico Hayes, Matthias Wissuwa

**Affiliations:** 1 Crop, Livestock and Environment Division, Japan International Research Center for Agricultural Sciences, Tsukuba, Ibaraki, Japan; 2 School of Biological Sciences, The University of Western Australia, Perth, Western Australia, Australia; Texas Tech University, UNITED STATES

## Abstract

Phosphorus (P) is an essential macronutrient for plant growth and development. Phosphorus is usually applied as fertilizer obtained from rock phosphate which is a non-renewable resource. Therefore, developing rice varieties that can use P more efficiently is crucial. Here, we investigated genotypic differences in traits related to internal Phosphorus Utilization Efficiency (PUE) in five rice genotypes grown under P-deficient conditions. P-efficient rice genotypes showed higher total biomass. This was partly due to higher root biomass, which in turn relied on preferential allocation of P to roots in these genotypes. Changes in P content and tissue P concentrations were analyzed in individual leaves at different time points. Genotypes belonging to the high-PUE group responded more quickly to P starvation in terms of reducing leaf P concentrations and they were able to reduce these concentrations to a lower level compared to the low-PUE group. Changes in P concentrations were reflected in gene expression levels for genes involved in lipid remodeling. Sulfolipid (*OsSQD2*) and galactolipid (*OsMGD* and *OsDGD*) synthesis-related genes were generally induced due to P starvation with most pronounced up-regulation in *OsDGD1* and *OsMGD3*, but patterns differed between genotypes. A significantly higher expression of *OsDGD5* and *OsMGD1 & 2* was detected in the youngest fully expanded leaf of the high-PUE genotype group, whereas expression levels were reversed in older leaves. This pattern would confirm that P efficient genotypes react faster to P starvation in terms of freeing P for redistribution to growing tissues and replacing phospholipids with galactolipids in younger leaves may contribute to this aspect.

## Introduction

Global food demand is predicted to double between 2000 and 2050 [1]. This poses a huge challenge for sustainable food production in terrestrial and aquatic ecosystems and their contribution to the world population [1]. Application of fertilizers to meet this demand is ever increasing. Nevertheless, the inefficient use of fertilizer causes the degradation of soil quality and eutrophication of aquatic habitats and adds costs to crop production for increased fertilization, irrigation and energy to sustain the production level in these degraded soils [2]. Pertinently, the global nitrogen (N) and phosphorus (P) fertilizer usage increased 7-fold and 3.5-fold, respectively, between the year 1960 and 1995. These fertilizer applications will increase by additional 3-fold by 2050, if efforts are not made to improve fertilizer efficiency in agriculture [3,4].

To tackle this problem that spans food production in both terrestrial and aquatic ecosystems, it will be imperative to significantly increase nutrient efficiency, which is possible through improved uptake of phosphate from soil (P-acquisition efficiency) [5] and improved biomass and/or yield per unit P taken up [internal P-utilization efficiency (PUE)] [6,7]. Less attention has been given to PUE and studies focused on PUE have often had the problem of confounding the variations due to P-acquisition efficiency [6,8,9]. Increasing P uptake can increase yield, however; it also increases the total amount of P removed from the field, ultimately causing P depletion. Increased P exports can cause considerable off-site environmental problems [10]. Sustainable and productive agricultural systems must have a balanced P export and input and high biomass or yield per unit P taken up and hence, high PUE is the clear preference [7]. Significant achievements have been made in terms of improved P-acquisition efficiency in crops, where a phosphate uptake gene (PUP1 QTL/*Pstol1*) was identified [11] and breeding effort is ongoing for the introgression of this gene into breeding lines, which is the first step in programs for improved P-efficiency [9]. Pyramiding of loci with P-uptake efficiency obtained from *Pup1* and loci identified from PUE will be highly indispensable for developing varieties that maintain high yield with low P-fertilizer input and also increase yields on vast rice cultivation areas with poor soil-P availability [9]. However, little progress has been made on PUE. Research efforts in dissecting the physiological, metabolic, molecular, genetic, and phylogenetic aspects of P-use efficiency is urgently needed for significant progress in understanding this complex trait [7] and the development of varieties with high P-use efficiency using this knowledge is of paramount importance. Therefore, our lab is leading efforts to develop P-efficient crops, both conceptually by dissecting P efficiency and indicating methods of analysis [8], and experimentally where the lab has identified candidate genes [9]. Several loci associated with PUE have been identified in different genetic backgrounds and efforts are underway to characterize these loci further, both genetically and physiologically. We have used natural rice genetic variation to investigate PUE of rice genotypes and their possible physiological and genetic control. Internal P utilization efficiency [defined here as the total biomass produced per unit P accumulated (g dry weight mg^-1^ P)] [6,8,9] was determined in plants grown under a controlled single P supply in hydroponics. Five rice genotypes with contrasting PUE were grown under P-stressed conditions for 50 days and growth, biomass, P concentration, P content, PUE and relative gene expressions of lipid metabolism-related genes were measured at up to three time points. High-PUE genotypes showed vegetative-vegetative P flux among leaves of different ages, as a physiological mechanism. In addition, high-PUE genotypes exhibited a reallocation of P from shoot to root (organ to organ) when P was limiting. This indicates a remobilization of P, as a P-efficiency trait. Genetic control of PUE was also investigated by characterizing the expression of PUE candidate genes across a time course and leaf age. High-PUE genotypes showed induction of galacto- and sulfolipid biosynthesis genes in younger leaves, which may replace phospholipids under P-stress, thus improving P-use efficiency [9,12–15].

## Materials and methods

### Plant material and growth conditions

Five rice (*Oryza sativa* L.) genotypes (DJ123 (DJ), Mudgo (MU), Yodanya (YO), IR64 (IR), and Taichung (TA)) were obtained from the breeding program at the Japan International Research Center for Agricultural Sciences (JIRCAS). Seeds of these varieties were washed three times by alcohol (70% v/v) and rinsed by deionized (DI) water and incubated for three days at 30°C for germination in Petri dishes. Germinated seeds were put in a mesh-tray on top of a 8-L plastic tub filled with DI water containing 100μM Ca^+2^ and 10μM Fe-EDTA in addition to a 1/20 (excluding N) Yoshida solution, added to prevent nutrient deficiency [16]. Following this 10-day pre-treatment period, 2 plants per genotype of equal size were transferred to a 1.1 L bottle filled with 1/10 strength Yoshida solution (excluding P). Phosphorus was supplied as a 1-time dose of 0.9 mg P. Every 3 days Yoshida solution (without P) was added as an equivalent of 1/10 strength (in 1.1L) and at these times the pH was adjusted to 5.8 using 50mM K_2_SiO_3_. Plants were grown in bottles for another 10 days to ensure the 1-time dose of P was fully taken up; after which they were transferred to 45L tubs with a capacity to carry 28 plants. After the plants were transferred to tubs, no P treatment was added, to keep the plants under P-deficient conditions. Other nutrients were supplied at ¼ strength Yoshida solution initially, and this increased to half-strength and full-strength Yoshida solution at 30 and 40 DAG, respectively. The plants were grown in the tubs until the final harvest at 50 DAG. The experiment was conducted in a controlled environment glasshouse between the month of September and November 2018. The photoperiod during the duration of the experiment was approximately 12 hr day/night. The temperature and relative humidity in the glasshouse were recorded with a data logger (Thermo Recorder TR-72Ui, T & D CORP, JAPAN). The maximum, minimum and average temperature were 35.1°C, 24.5°C, and 28.0°C, respectively and the average relative humidity was 32.6%. The experiment was arranged in a completely randomized design with four replications and two plants per bottle/tub slot. The sampling time and type of sampling is presented in S1 Fig.

### Agronomical and physiological measurements

Leaf number was counted at 20, 40, and 50 DAG to record leaf development across time. Plant height and root length was measured at the end of the experiment using a ruler. Roots were separated from shoots and shoots were divided into the emerging leaf (L0), the youngest fully expanded leaf (L1), the subsequent leaves L2 and L3, and the remaining leaves and stem. Sampling were done at three time points [20 DAG (T1), 40 DAG (T2), 50 DAG (T3)]. Plant material was oven-dried at 70°C for three days and dry weight (Dwt) was recorded. Total dry weight was calculated as the sum of shoot and root Dwt. Root to shoot ratio (root:shoot) was calculated as root Dwt/shoot Dwt. In addition, some individual leaves were sampled at 20 DAG (L1), 40 DAG (L1 and L2), 50 DAG (L0, L1, L2, L3); (S1 Fig).

### Phosphorus concentration measurement

Phosphorus concentration of leaf and root tissues were measured by wet-acid digestion in 6 mL of a 3:1 HNO_3_/HClO_4_ mixture. Digests were diluted to a total volume of 50ml with DI water. Phosphorus concentration analysis was done using Malachite Green colorimetric P analysis method [17]. The shoot and root P content was calculated by multiplying the P concentration with shoot and root biomass. Total P content was calculated as the sum of shoot and root P content. P concentration and content were analyzed for 3 time points T1, T2 and T3 and within these time points for L1 at T1, L1 & L2 at T2 and L0, L1, L2, L3 at T3 where T stands for time and L stands for leaf.

### Expression analysis for PUE candidate genes

Expression of lipid remodeling-related genes (*OsSQD2*, *OsMGD1*-*3*, *OsDGD1*-*5*) were tested on five rice genotypes (MU, DJ, YO, TA, & IR) across three time points 20, 40 & 50 DAG (designated as T1, T2 & T3). L1 was collected at T1, L1 and L2 were collected at T2, and L0, L1, L2, L3, and roots (R) were collected at T3. Samples were flash frozen in liquid nitrogen and stored at -80°C until the analysis. RNA was isolated using RNA isoPlus (Cat. #9109) (Takara Bio) and reverse-transcribed using Prime Script RT Master Mix (Perfect Real Time) (Takara Bio). QPCR was done using TB Green Premix Ex Taq II SYBR Green master mix (Takara Bio) and gene-specific primers (S1 Table) and run in CFX96TM Real-Time System (Bio Rad). QPCR conditions were 95°C 30 s; 95°C 5 s, 60°C 30 s for 40 cycles. The expression level of each gene was quantified with a standard curve, using *OsC3H38* as the internal reference gene [18].

### Statistical analysis

Data analysis was done using R statistical software [19]. The significant differences between means were determined using ANOVA in conjunction with the LSD test at the P<0.05 level of significance. All data shown are means ± SE. For statistical analysis, agricolae package version: 1.3–1 was used [20]. The standard error was computed using ddply/plyr: split data frame, apply function, and return results in a data frame [21]. To visualize data, ‘tidyverse’ package version 1.3.0 was used and under this package ggplot2 version 3.2.1 was used [22]. Correlation was done using the ggpubr R package for an easy ggplot2-based data visualization (http://ggplot2.tidyverse.org), corrplot package to plot correlograms [23,24], Hmisc to calculate correlation matrices containing both correlation coefficients and p-values [25], and tidyverse for all the data wrangling, plotting and alike. Pearson correlation (r), which measures a linear dependence between two variables (x and y) was employed and it depends to the distribution of the data. It can be used only when x and y are from normal distribution [23,24].

## Results

### Growth and biomass

**Fig 1:**
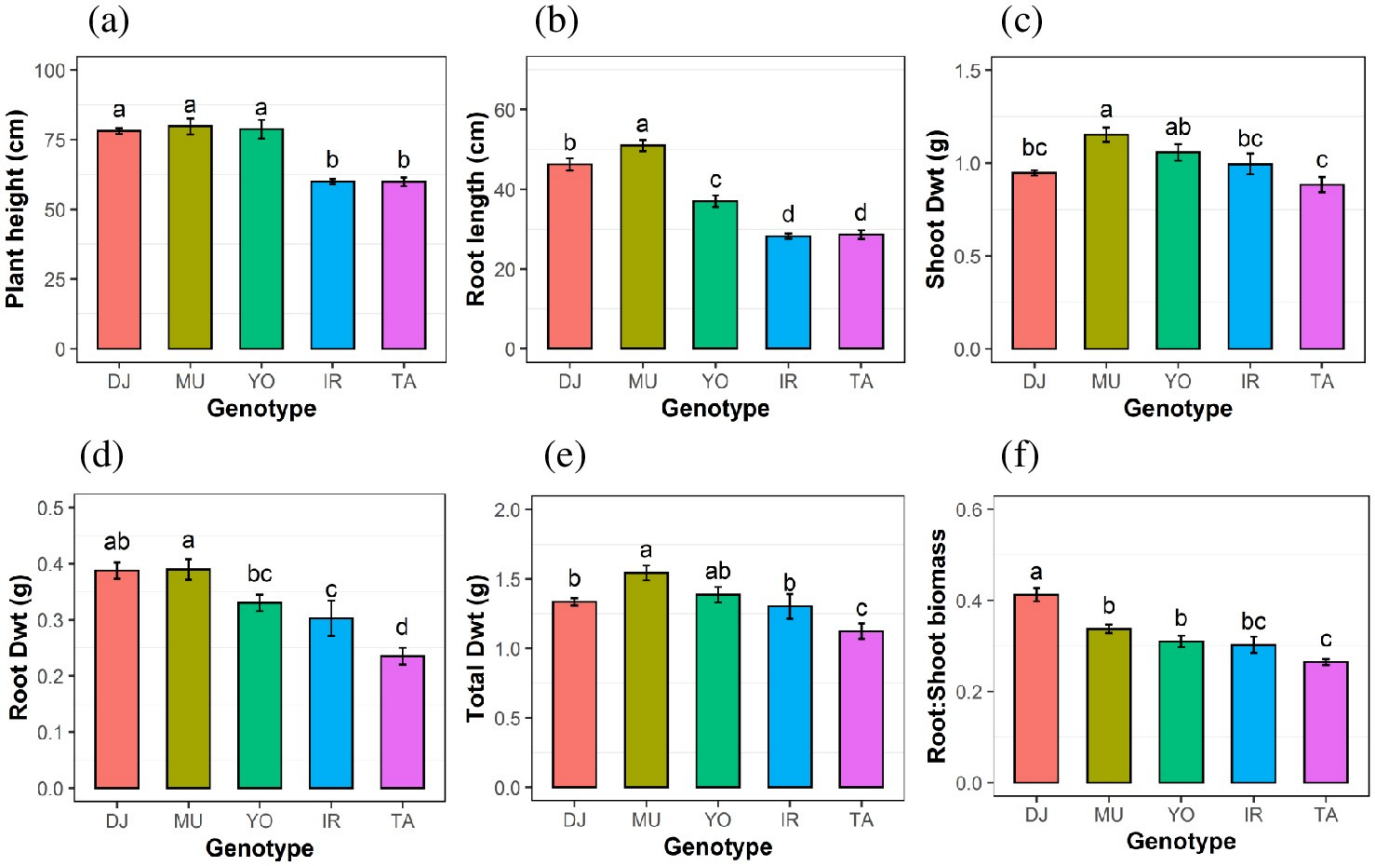
Differences in growth among genotypes. Plant height (a), root length (b), shoot dry weight (Dwt) (c), root Dwt (d), whole plant Dwt (e), and root to shoot Dwt ratio (f), of five rice genotypes after 50 days of growth in phosphorus deficient nutrient solution. Mean ± S.E (n = 4). Different letters above bars indicate significant differences among genotypes (LSD, p ≤ 0.05).

Phosphorus was supplied as a single dose of 0.9 mg P at 10 days after germination (DAG) (see M&M and S1 Fig) and that provided sufficient P for normal plant growth up to the first sampling time point at 20 DAG (T1), whereas plants should have become P deficient by time points 40 DAG (T2) and 50 DAG (T3). Plant height differences were pronounced at 50 DAG with genotypes DJ123, Mudgo and Yodanya almost 20 cm taller compared with IR64 and Taichung (Fig 1a). A similar ranking was observed for maximum root length, with longest roots in Mudgo (52.8cm), followed by DJ123 (46.3cm) and Yodanya (37 cm), while Taichung and IR64 had root lengths of only 28.6 cm and 28.3 cm, respectively (Fig 1b). Leaf number was assessed at all three time points and IR64 tended to have the highest leaf number, followed by Taichung (S2 Fig) with Mudgo the lowest. Leaf number more than doubled, from 3–4 at 20 DAG, to 7–9 leaves per plant at 40 DAG; thereafter leaf development slowed down.

Highest shoot dry weight was reached by Mudgo, followed by Yodanya, IR64, DJ123, and Taichung (Fig 1c). Mudgo also had the highest root dry weight, with DJ123 being a close second and Taichung being the lowest (Fig 1d). As a result, the highest total biomass of 1.6 g per plant was reached by Mudgo, followed by Yodanya, DJ123, IR64 (all between 1.3–1.5g), and Taichung, in that order (Fig 1e). The root to shoot biomass ratio was highest in DJ123 (0.42) followed by Mudgo (0.34), IR64 and Yodanya (~0.3). Taichung had the lowest root to shoot ratio (0.28) (Fig 1f).

### Phosphorus concentration and internal phosphorus-utilization efficiency

**Fig 2:**
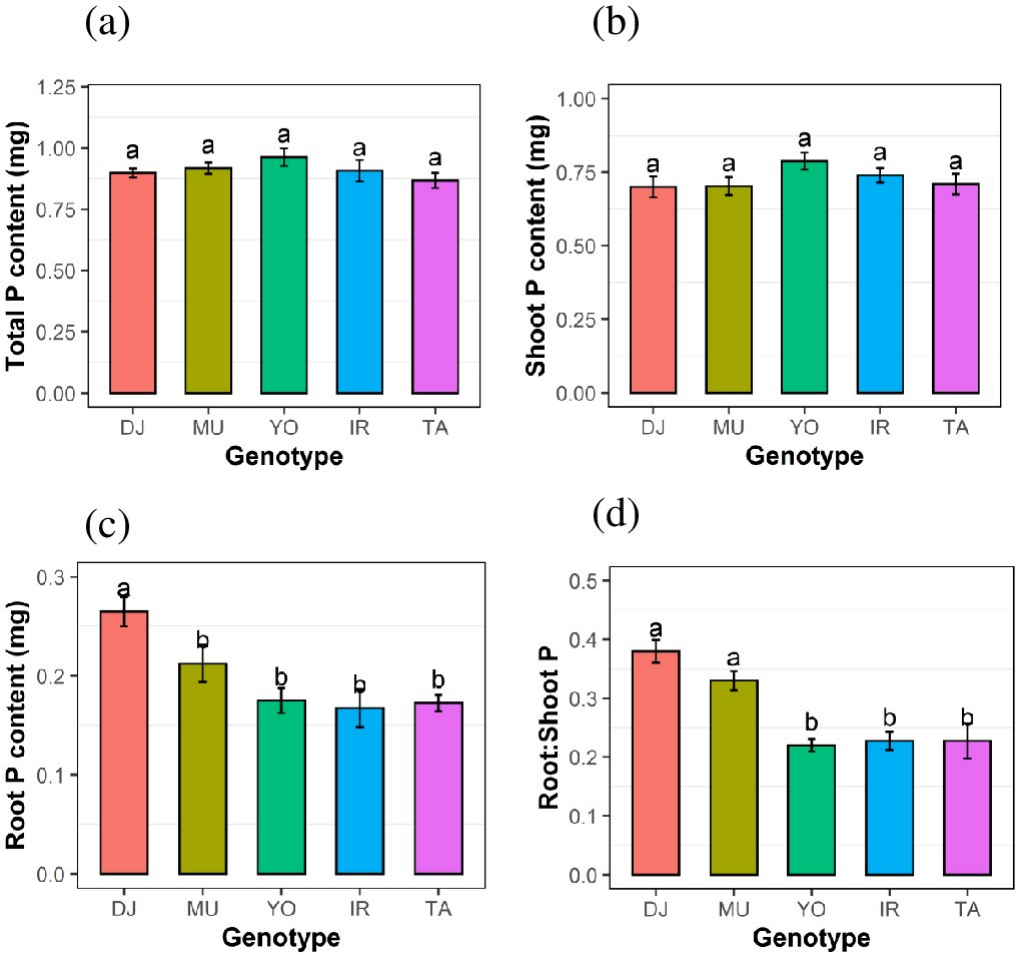
Phosphorus (P) content among genotypes. Whole plant (a), shoot (b), and root (c) P contents, as well as root to shoot P content ratio (d), of five rice genotypes after 50 days of growth in phosphorus deficient nutrient solution. All values are per bottle, with each bottle containing two plants. Mean ± S.E (n = 4). Different letters above bars indicate significant differences among genotypes (LSD, p ≤ 0.05).

**Fig 3:**
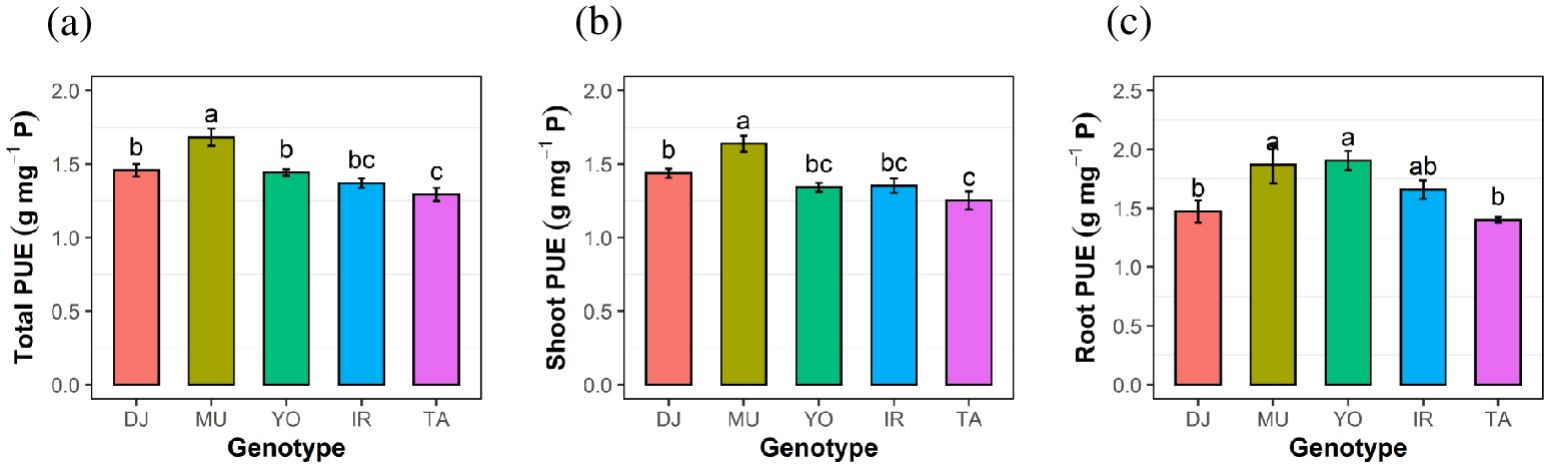
Internal phosphorus (P) utilization efficiency (PUE). Whole-plant (a), shoot (b), and root (c) PUE, of five rice genotypes after 50 days of growth in P deficient nutrient solution. Mean ± S.E (n = 4). Different letters above bars indicate significant differences among genotypes (LSD, p ≤ 0.05).

All genotypes were supplied with the same quantity of P (0.9 mg P per bottle containing two plants) and total P content therefore did not differ among genotypes (Fig 2a); neither did shoot P content (Fig 2b). This allowed us to evaluate physiological PUE (estimated as tissue biomass per amount of P in respective tissue) at equal P content. Genotypic differences in PUE were significant, with Mudgo being the most efficient, followed by DJ123 and Yodanya (Fig 3a). Mudgo and Yodanya also had the highest root PUE (Fig 3c) while low root PUE of DJ123 was likely due to this genotype’s higher root P content (Fig 2c).

### Changes in leaf phosphorus concentrations over time

**Fig 4:**
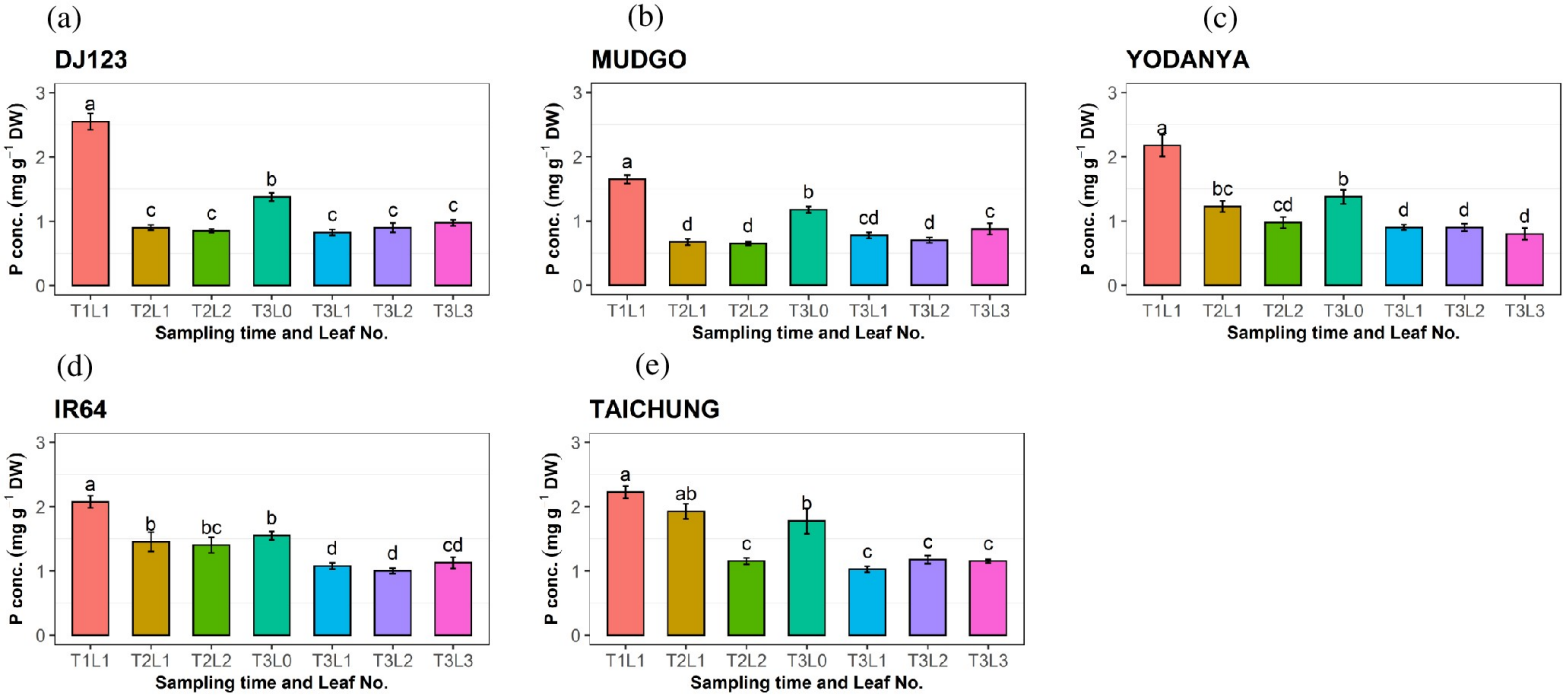
Individual leaf phosphorus (P) concentrations for each genotype. Leaf P concentrations of differently aged leaves collected at three sampling times, for the five rice genotypes DJ123 (a), Mudgo (b), Yodanya (c), IR64 (d), and Taichung (e). Plants were grown for 50 days in phosphorus deficient nutrient solution and sampled at three-time points: 20 DAG (T1), 40 DAG (T2) and 50 DAG (T3). Different leaf stages were sampled: L0 indicates not yet fully expanded leaf, L1 represents the youngest fully expanded leaf, L2 and L3- the 2nd and 3rd leaf from the top. Mean ± S.E (n = 4). Different letters above bars indicate significant differences among the leaves for each genotype (LSD, p ≤ 0.05).

**Fig 5:**
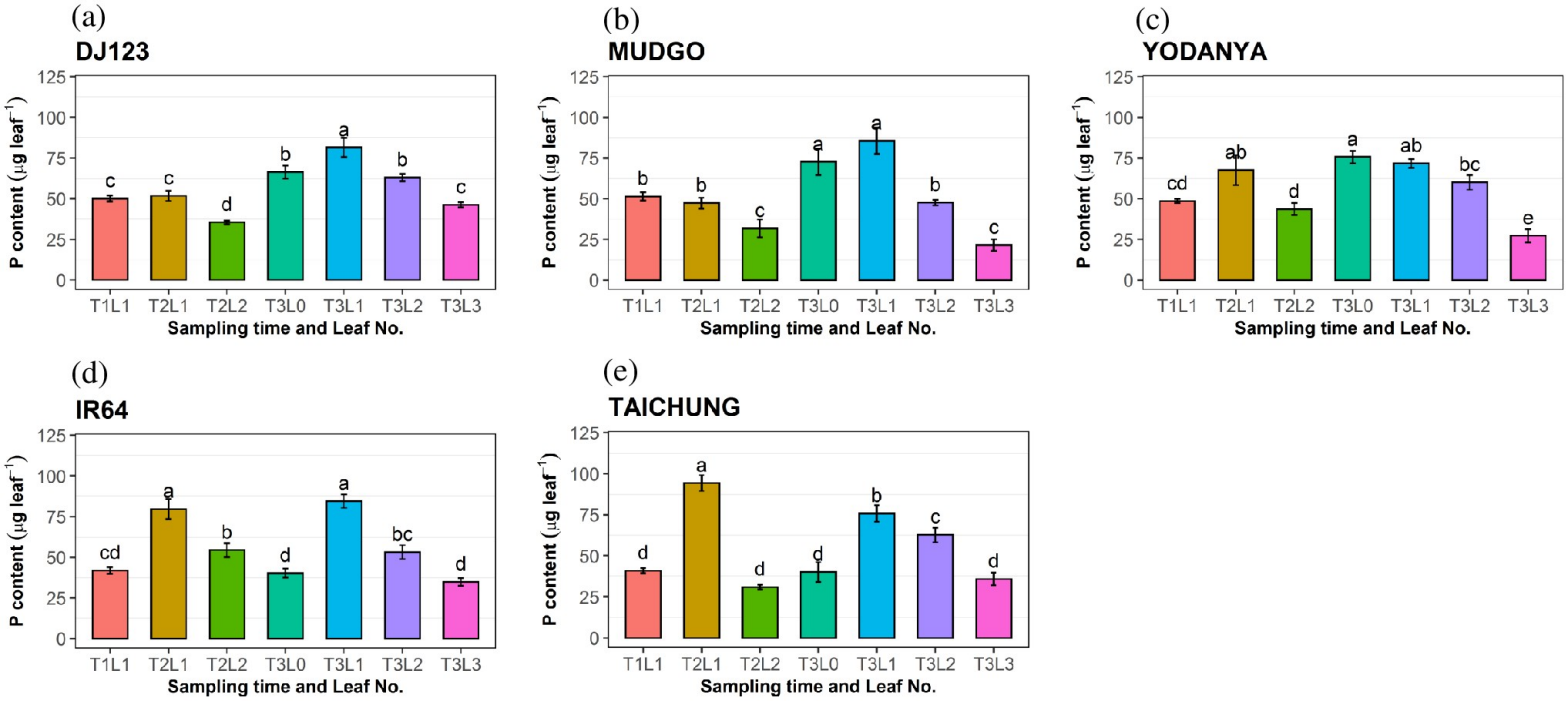
Individual leaf phosphorus (P) contents for each genotype. Leaf P contents of differently aged leaves collected at three sampling times, for the five rice genotypes DJ123 (a), Mudgo (b), Yodanya (c), IR64 (d), and Taichung (e). Plants were grown for 50 days in phosphorus deficient nutrient solution and sampled at three-time points: 20 DAG (T1), 40 DAG (T2) and 50 DAG (T3). Different leaf stages were sampled: L0 indicates not yet fully expanded leaf, L1 represents the youngest fully expanded leaf, L2 and L3- the 2nd and 3rd leaf from the top. Mean ± S.E (n = 4). Different letters above bars indicate significant differences among the leaves for each genotype (LSD, p ≤ 0.05).

Changes in leaf P concentration were assessed over time (Fig 4). At 20 DAG (T1; see M&M) plants did not appear P deficient and leaf P concentrations in T1L1 (see M&M) were mostly above 2 mg g^-1^ (the concentration considered adequate for crop growth, [26]). After 20 more days of growth without further P addition (40 DAG; T2), leaf P concentrations had decreased in T2L1 and T2L2 and more so in the high-PUE genotypes DJ123 and Mudgo, compared with the low-PUE genotypes IR64 and Taichung (Fig 4). After a further 10 days (50 DAG; T3), leaf P concentrations tended to decrease further in the low-PUE genotypes, reaching a similarly low leaf P concentration compared with the high-PUE genotypes at 40 DAG. An exception was the expanding leaf (T3L0), which maintained higher P concentrations compared to other leaves in all genotypes (Fig 4). The corresponding leaf P contents (leaf P content per single leaf) were also determined over time (Fig 5). The most distinct difference between high-PUE and low-PUE genotypes was the very high P content in the youngest leaf (T2L1) at 40 DAG, seen in IR64 and Taichung, which were between 75–100 μg P, compared with ~50 μg P in DJ123 and Mudgo. This was due to pronounced differences in the P concentration in this leaf class between groups: Mudgo and DJ123 had produced this youngest leaf (T2L1) with P concentrations below 1 mg g^-1^ (Fig 4a and 4b), while these were ≥ 1.5 mg g^-1^ in IR64 and Taichung (Fig 4d and 4e).

### Expression of lipid-synthesis related genes in contrasting PUE groups

Phospholipids are an important reserve for P, but under P-deficient conditions they can be substituted by other classes of lipids, such as sulfolipids and galactolipids, which is suggested to facilitate more efficient internal utilization of P [13]. We hypothesized that high-PUE genotypes more strongly and/or rapidly induce the expression of genes involved in sulfolipid and galactolipid synthesis during P starvation, to free P for other uses. Thus, we collected leaf tissues at different time points (T1, T2, and T3) and analyzed the expression of genes related to the synthesis of these lipids. At T3, we additionally analyzed the expression levels in root tissue, since different patterns of lipid remodeling are triggered in root and shoot [13].

**Fig 6:**
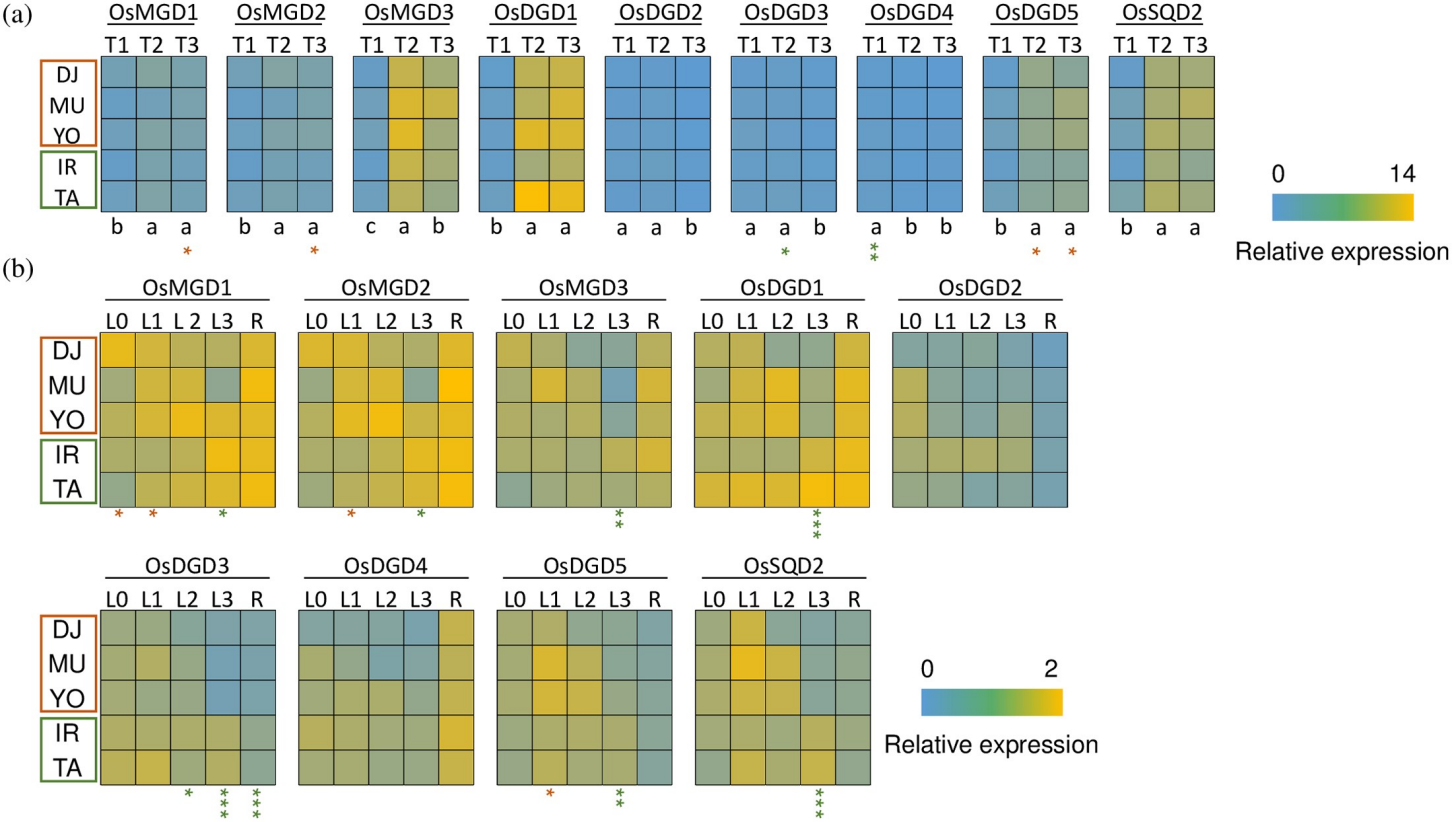
Expression of lipid synthesis-related genes among five contrasting genotypes. Comparison of expression level at different time points (T1, T2 and T3) in the youngest fully expanded leaf (L1) (a). Comparison of the expression level in different tissues (L0, L1, L2, L3 and root [R]) at T3 (b). Plants were grown for 50 days in phosphorus deficient nutrient solution and sampled at three-time points: 20 DAG (T1), 40 DAG (T2) and 50 DAG (T3). Different leaf stages were sampled: L0 indicates unexpanded leaf, L1 represents the youngest fully expanded leaf, L2 and L3- the 2nd and 3rd leaf from the top. Mean values were determined for each gene and sample group, and relative expression levels are shown by heat plot. In (a), the expression at T1 in IR was considered as 1. In (b), the expression in L1 in IR was considered as 1. The expression level of each gene was compared between the high-PUE group (DJ, MU, and YO; indicated by red square) and the low-PUE group (IR and TA; indicated by green square) by two-tailed Student’s t-test. Significant differences are indicated as asterisks as follows: *, p < 0.05; **, p < 0.01; ***, p < 0.001. Red and green asterisks indicate higher expression in high-PUE group and low-PUE group, respectively.

Since the content of sulfolipid (sulfoquinovosyldiacylglycerol) and galactolipids (monogalactosyldiacylglycerol and digalactosyldiacylglycerol) increases strongly under P starvation [13], we selected OsSQD2 (sulfoquinovosyldiacylglycerol synthase 2), OsMGD1-3 (monogalactosyldiacylglycerol synthase 1–3), and OsDGD1-5 (digalactosyldiacylglycerol synthase 1–5) as potential candidate genes positively affecting PUE. Phosphorus deficiency strongly induced the expression of *OsMGD3*, *OsDGD1* (>3-fold) and in *OsDGD5 and OsSQD2* (2-fold), whereas the upregulation was weak in *OsMGD1* and *2* (Fig 6a). *OsDGD2-4* were not upregulated under P starvation, suggesting that these genes do not play a crucial role in P deficiency-mediated lipid remodeling. Genotypic differences in gene expression between genotype groups were detected in the youngest fully expanded leaf for *OsDGD5* and *OsMGD1* and *2*, with consistently higher expression in the high-PUE genotypes at T3 (Fig 6b). The contrast of *OsDGD5* and *OsMGD1* and *2* observed in L1 was not found in other tissues. However, in L3 consistently lower expression in high-PUE genotypes were detected for several genes, which might be associated with accelerated senescence in this leaf in high-PUE genotypes.

## Discussion

Improving the efficiency of phosphate utilization in agriculture has been a goal in light of protecting the environment as well as reducing input costs and delaying the exhaustion of non-renewable rock phosphate resources [27,28]. However, research efforts have primarily focused on P-uptake efficiency [5,11]. Because improving uptake efficiency alone will deplete the soil of available P in low input agriculture and increase input cost in high input agriculture, there is a growing interest to improve internal P-utilization efficiency (PUE) in crop plants [6,7].

Previous work in our group highlighted the confounding effect of P uptake on PUE and devised a screening method that avoids this problem by supplying equal amounts of P to each plant or genotype [9]. Internal P-utilization efficiency is then estimated based on biomass accumulated per P acquired. The current experiment adopted this methodology and consequently all rice genotypes used in this experiment showed an equivalent total P content in the whole plant (Fig 2a). Therefore, it was possible to obtain estimates of PUE not confounded by differences in the level of P starvation caused by differences in P uptake.

The high-PUE genotypes maintain lower P concentrations in leaves developed under P deficient conditions. Furthermore, there seemed to have been a difference between low-PUE and high-PUE genotypes in terms of where P was allocated. Low-PUE genotypes IR64 and Taichung initially maintained high P concentrations in young leaves, developing under P deficiency (T2L1) and we therefore suggest that these developing leaves were a strong sink for P, at least during early stages of P deprivation. The high-PUE genotypes Mudgo and DJ123 on the other hand showed very low P concentrations and P content in the youngest leaf, even at 40 DAG, suggesting that the P may have been distributed to other plant organs. The higher proportion of P allocated to roots in these genotypes (Fig 2d) may indicate that roots were competing successfully with developing young leaves as a sink for P during P deficiency.

Any potentially preferential allocation to roots rather than developing leaves in the high-PUE genotypes did, however, not reduce the biomass of leaves in these genotypes in comparison to the low-PUE genotypes (S3 Fig). Thus, the high-PUE genotypes appeared to more rapidly reduce P concentrations in newly developed and older leaves alike, and likely allocated the extra P to root growth, which would be an efficient strategy to outgrow P deficiency as new roots will increase the amount of P taken up by the plant [29,30] investigated to what extent reduced leaf P concentrations affect leaf photosynthetic efficiency. At the range of P concentrations seen here at T2 in the high-PUE genotypes (< 1.0 mg g^-1^) one may expect reductions in CO_2_ assimilation rates by up to 30% and these will be less in IR64 and Taichung with leaf P concentrations well-above 1.0 mg g^-1^. While this can be compensated to some extent in high-PUE genotypes by producing larger leaves [30], we may expect the loss of photosynthetic capacity to be more pronounced in the high-PUE genotypes. In this regard one needs to keep in mind that the strategy to preferentially supply P for root growth does not provide additional P uptake in this nutrient solution experiment, while it would do so in plants grown in soil. This additional P uptake would allow plants to increase their leaf P concentrations [29], thus avoiding prolonged losses in carbon assimilation.

One of the mechanisms to use P efficiently is to change the lipid composition of the plant [31], which helps the plant to utilize 15–30% of the lipid-bound P in the cell [32]. Lipid remodeling during P starvation is termed as the change of lipid composition by degrading phospholipids and synthesizing glycolipids (including sulfolipids and galactolipids) [13]. Phosphorus starvation induces the expression of SQDG genes (SQD1 and 2) [33,34], which are genes encoding enzymes that synthesize the sulfolipid head group and then add the head group to diacylglycerol (DAG), respectively. Contrary to our hypothesis, the up regulation of *OsSQD2* appears a general phenomenon in rice not linked to genotypic differences in efficient P translocation. On the other hand, we observed different expression patterns of *OsMGD 1* and *2* and *OsDGD5* in high- and low-PUE groups. Faster or stronger induction of these genes in high-PUE genotypes may explain more efficient P reallocation from L1 during P starvation, which is consistent with our P concentration analysis. It was interesting to note that gene expression patterns reverse in older leaves. For seven out of nine genes higher relative expression was detected in the low-PUE group (Fig 6). Thus, it was not the absolute ability to regulate lipid remodeling genes that differed between groups but the onset of such regulation, which was significantly earlier in times of leaf age in the high-PUE group. In case of *OsMGD1* higher expression in the high-PUE group was already detected in the developing leaf, possibly suggesting that galactolipids may substitute phospholipids already during organogenesis in P efficient genotypes. The regulatory factors for these genes, as well as actual changes in lipid composition and in gene expression of phosphatases involved in phospholipid breakdown [12,27] warrants further investigation.

Phosphorus-starvation triggered lipid remodeling is well studied, the regulation of lipid remodeling however has remained elusive until recently [13]. The well-known transcription factor that regulates P starvation response is the MYB family factor PHR1 [35,36]. PHR1 together with microRNA399 (miR399) and PHO2, an E2 ubiquitin-conjugase, constitutes a systemic signaling pathway that communicates shoot Pi status to the root [37,38]. The loss of PHR1 protein under P starvation attenuated the decrease of phospholipids and the accumulation of MGDG and SQDG in shoots and roots [13]. Furthermore, [9,14] also discussed the degradation of phospholipids and the synthesis of sulfolipids as a P-stress induced mechanism that contributes to improved PUE. The above evidence corroborates the remobilization of P from leaf tissue, as indicated in our study.

The total dry weight produced under P deficiency was closely correlated with overall PUE (r = 0.81) and this is to be expected as were the close correlations between both traits and dry weights and shoot and root PUE (S4 Fig). The positive correlation of PUE and total dry weight (TDW) with root to shoot ratio (0.48 and 0.45, respectively) indicated that maintaining relatively more root growth was a positive attribute. This contrasted with the negative correlation between PUE and TDW with leaf number (-0.69 and -0.63, respectively; S4 Fig). Thus, having more leaves, which was observed in low-PUE genotypes (S2 Fig), leads to less efficient utilization of P. This may reflect the relatively high cost newly developing leaves incur in terms of P allocation, which happens at shorter intervals with more rapid leaf emergence. It may therefore be more efficient to develop fewer but larger leaves [30]. It should be noted that the low-PUE genotypes are modern semi-dwarf rice varieties whereas the high-PUE group consisted of traditional varieties characterized by being generally more vigorous [8]. To what extent general vigor would contribute to improved PUE under conditions where one nutrient such as P is clearly limiting remains to be elucidated and differences in rates of new leaf emergence *versus* leaf expansion should be considered further in this regard.

In addition, accelerated leaf senescence is known to be an important factor for PUE [39], however, our data does not indicate a decline in P concentration in older leaves (T3L3) of high-PUE genotypes, but rather a consistent lower P concentration across all mature leaves (Fig 4). Gene expression data nevertheless provides some indirect hints that senescence may have differed between groups of genotypes. In T3L3 samples seven out of nine genes showed higher relative expression in the low-PUE group, which therefore seems to be actively remodeling lipids in L3 at that time. The high-PUE group on the other hand showed low gene activity in T3L3 but much higher activity in younger leaves (Fig 6). Such low activity may indicate that biosynthetic activities are being replaced by catabolic processes as one would expect to occur during senescence.

In previous work, it was shown that one of the successful second generation green revolution rice varieties (IR64) lacks two of the efficient haplotype identified on chromosome 1, 4, & 12 and accessions with all the three favorable haplotypes include Mudgo and Yodanya which has an implication that there is an opportunity for the introgression of these PUE favorable haplotypes to such elite cultivars [9]. One additional aspect identified here is related to the factor of time. The largest differences between high-PUE and low-PUE genotypes were identified at 40 DAG, where high PUE genotypes DJ123 and Mudgo already reduced leaf P concentrations to the same low level detected at 50 DAG (Fig 4). IR64 on the other hand maintained high leaf P concentrations at 40 DAG but significantly reduced it at 50 DAG. This observation highlights the importance of time in the response to P deficiency, both in terms of identifying divergent time points between genotypes, as well as in formulating hypotheses regarding the molecular mechanisms of sensing and responding to P deficiency.

## Supporting information

S1 Fig Experimental time frame and sampling time layout. (TIF)

S2 Fig Number of leaves for each genotype across time. Leaf number at three vegetative growth stages for five rice genotypes during 50 days of growth in phosphorus deficient nutrient solution. Mean± S.E (n = 4). Different letters above points indicate significant differences among genotypes (LSD, p ≤ 0.05).
(TIF)

S3 Fig Individual leaf dry weight of rice genotypes. Individual leaf dry weights of DJ123 (a), Mudgo (b), Yodanya (c), IR64 (d), and Taichung (e) after 50 days of growth in phosphorus deficient nutrient solution and sampled at three time points at 20 DAG (T1), 40 DAG (T2) and 50 DAG (T3). Different leaf stages were sampled: L0 indicates not yet fully expanded leaf, L1 represents the youngest fully expanded leaf, L2 and L3- the 2nd and 3rd leaf from the top. Mean± S.E (n = 4). Different letters above bars indicate significant differences between genotypes (LSD, p ≤ 0.05).
(TIF)

S4 Fig Pearson correlation (r) for 13 traits. Correlation r-values listed for the traits (a), positive genetic correlations are in blue and negative correlations are in red (b). In panel b, the scale -1 to 1 indicates negative to positive correlation and the color intensity indicates the strength of the correlation.
(TIF)

S1 Table Primers used in the study. (XLSX)

S1 Appendix Phenotypic data. (XLSX)

S2 Appendix Gene expression data (qPCR). Data presented is on a linear scale using the standard curve method with the internal reference gene OsC3H38 (Os05g0564200).
(XLSX)
