# Supplementary figures and images for: Genetic and physiological traits for internal phosphorus utilization efficiency in rice
Source: PLoS One. 2020 Nov 5;15(11):e0241842. doi: 10.1371/journal.pone.0241842 (PMC7644049; doi:10.1371/journal.pone.0241842)

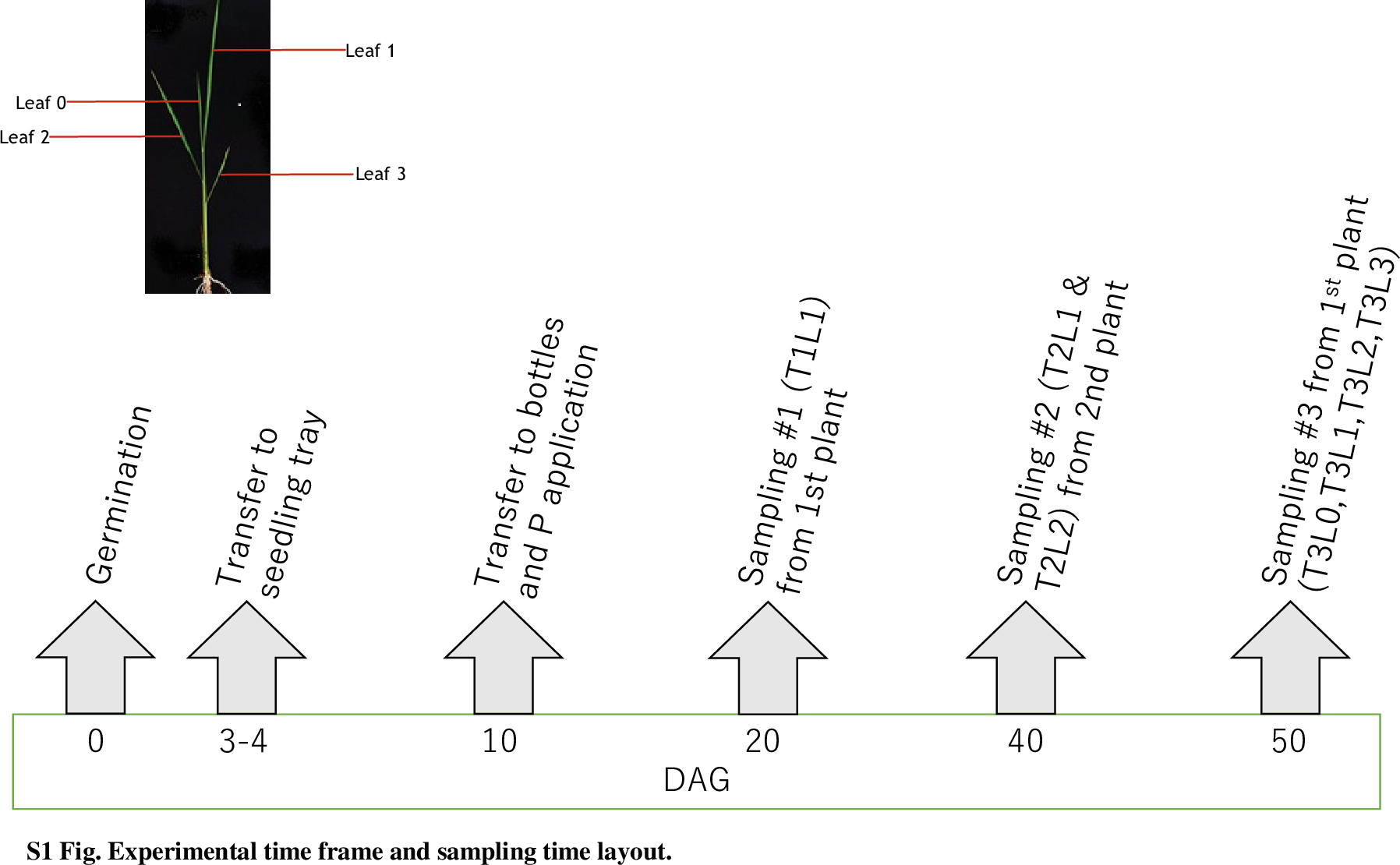

Supplement: S1 Fig — (TIF) [file pone.0241842.s001.tif]

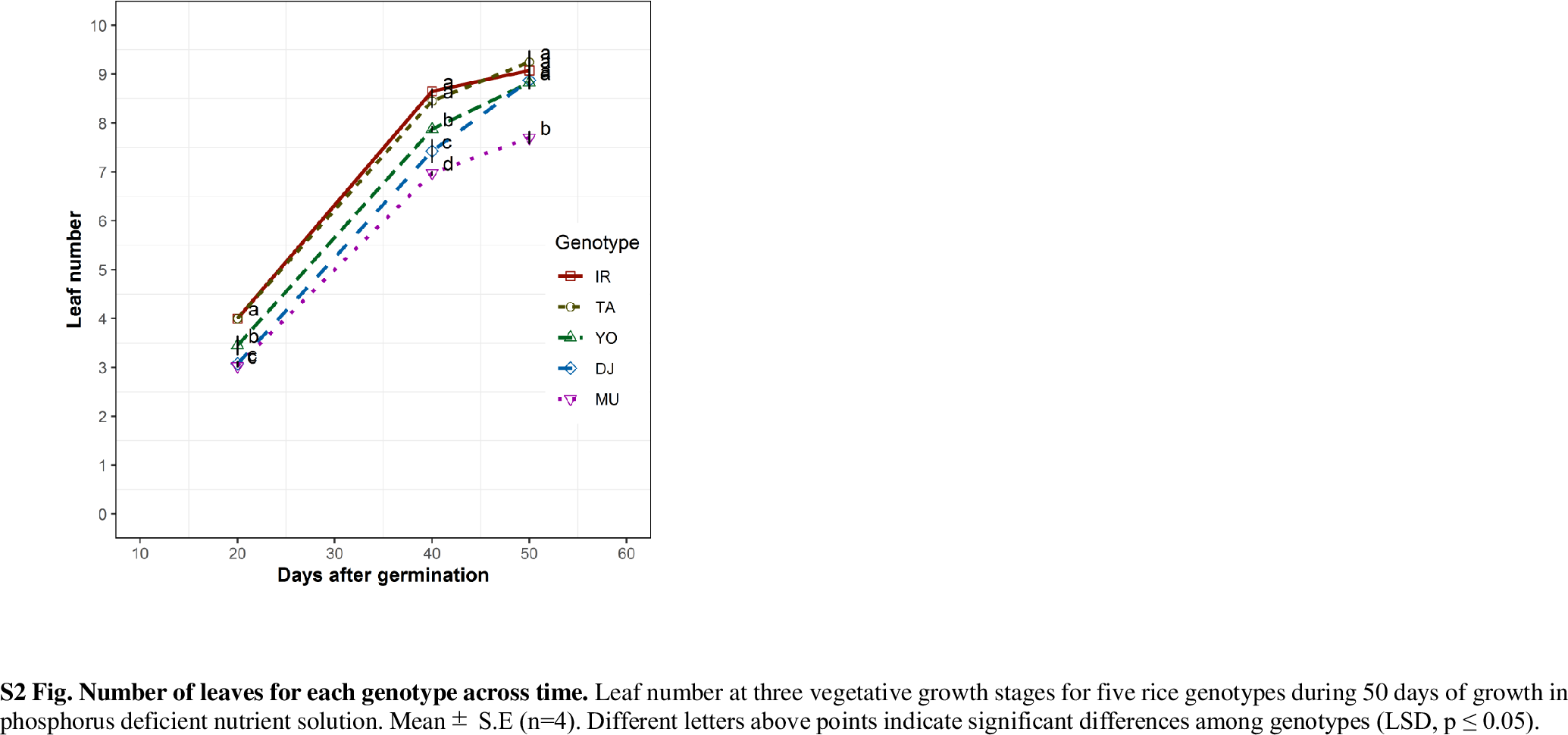

Supplement: S2 Fig — Leaf number at three vegetative growth stages for five rice genotypes during 50 days of growth in phosphorus deficient nutrient solution. Mean± S.E (n = 4). Different letters above points indicate significant differences among genotypes (LSD, p ≤ 0.05). (TIF) [file pone.0241842.s002.tif]

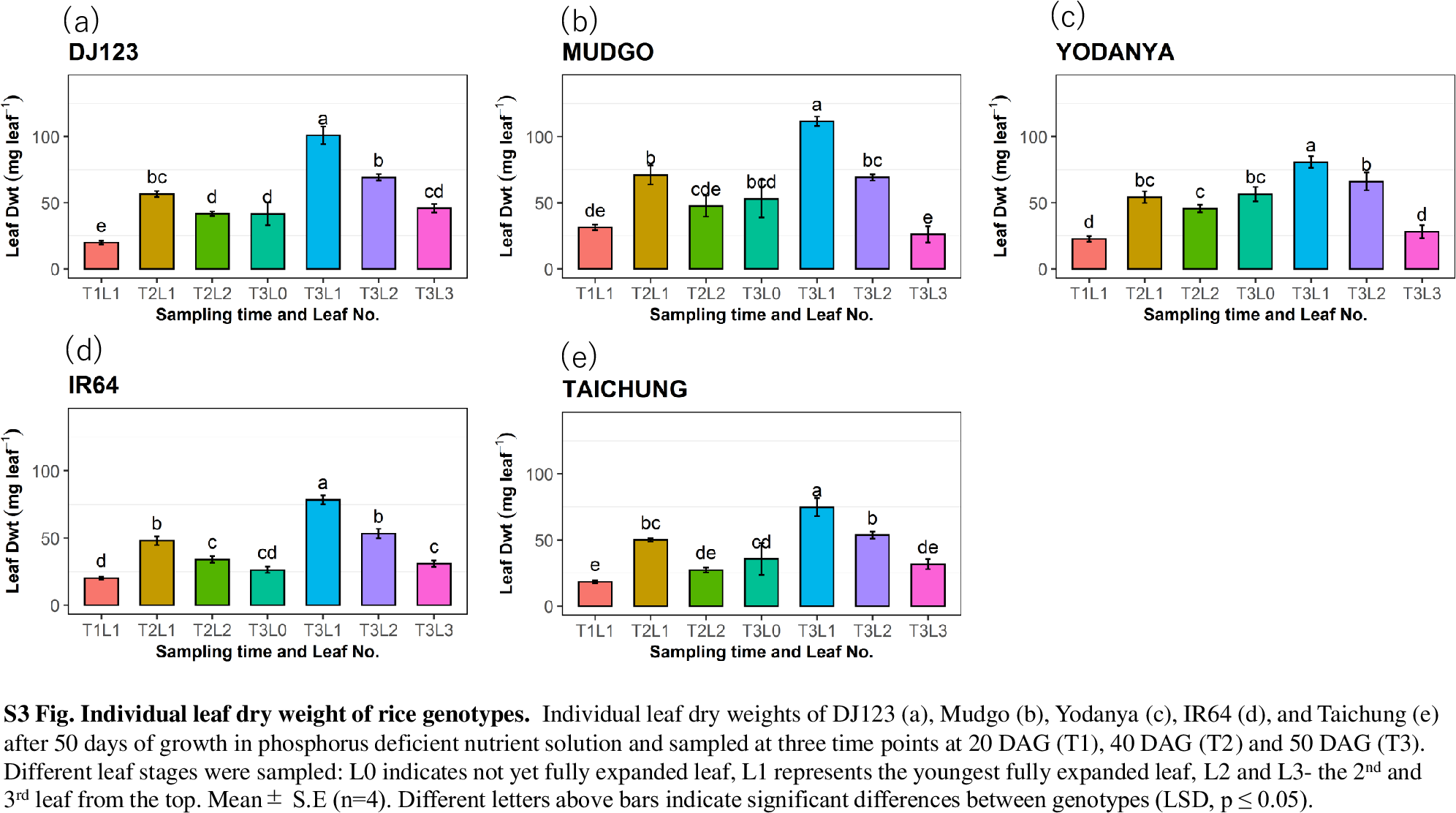

Supplement: S3 Fig — Individual leaf dry weights of DJ123 (a), Mudgo (b), Yodanya (c), IR64 (d), and Taichung (e) after 50 days of growth in phosphorus deficient nutrient solution and sampled at three time points at 20 DAG (T1), 40 DAG (T2) and 50 DAG (T3). Different leaf stages were sampled: L0 indicates not yet fully expanded leaf, L1 represents the youngest fully expanded leaf, L2 and L3- the 2nd and 3rd leaf from the top. Mean± S.E (n = 4). Different letters above bars indicate significant differences between genotypes (LSD, p ≤ 0.05). (TIF) [file pone.0241842.s003.tif]

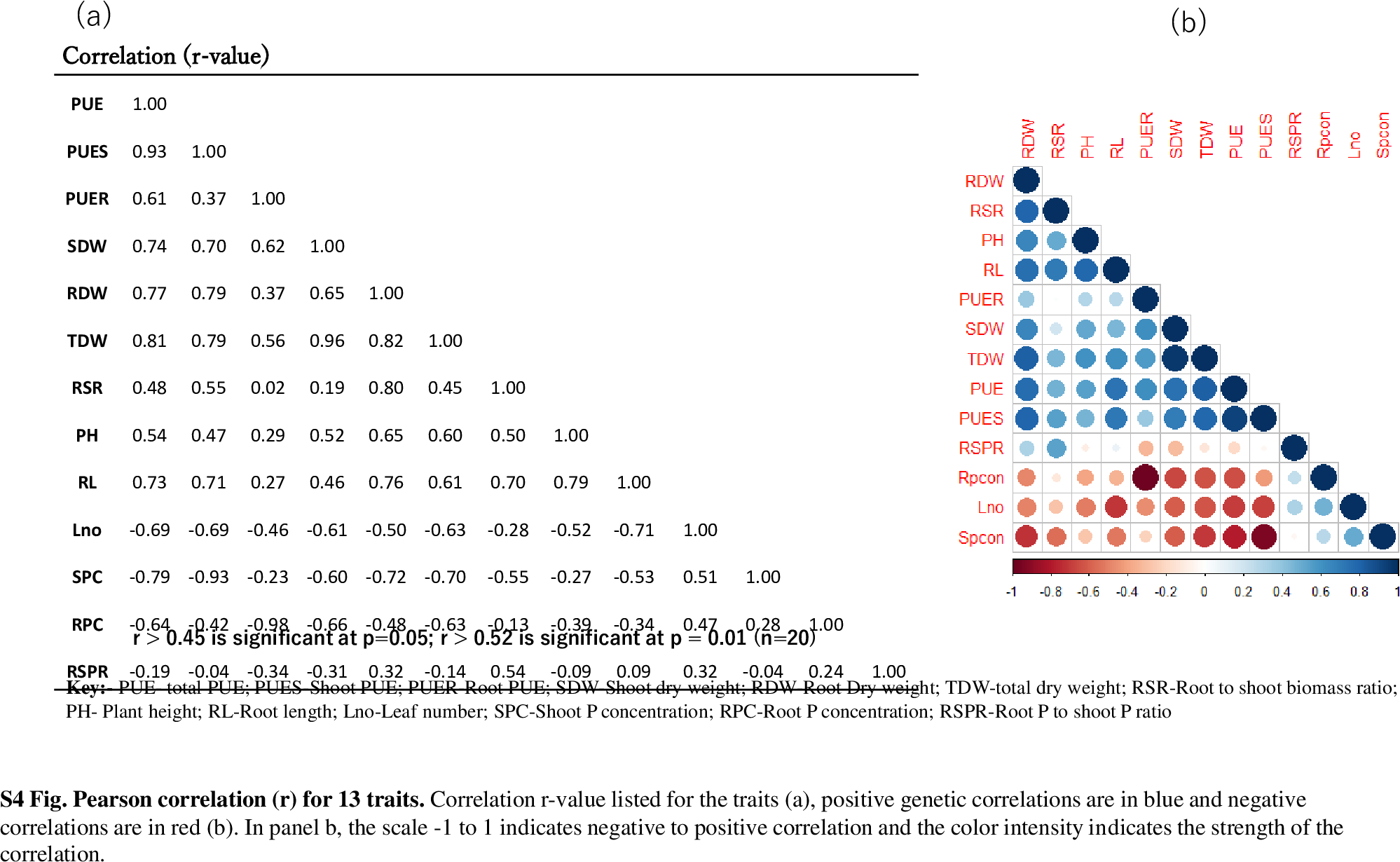

Supplement: S4 Fig — Correlation r-values listed for the traits (a), positive genetic correlations are in blue and negative correlations are in red (b). In panel b, the scale -1 to 1 indicates negative to positive correlation and the color intensity indicates the strength of the correlation. (TIF) [file pone.0241842.s004.tif]
